# South Asia symposium on pneumococcal disease and the promise of vaccines – Meeting report

**DOI:** 10.1016/j.vaccine.2016.03.071

**Published:** 2016-05-17

**Authors:** Rakesh Kumar, Narendra Arora, Mathuram Santosham

**Affiliations:** a Reproductive & Child Health Programme, Ministry of Health and Family Welfare, New Delhi, India; b INCLEN Trust International, New Delhi, India; c International Vaccine Access Center, Department of International Health, Johns Hopkins Bloomberg School of Public Health, Baltimore, USA

**Keywords:** *Streptococcus pneumoniae*, Pneumococcal conjugate vaccines, Immunization programs, South Asia, India, Pneumococcal disease burden

## Abstract

Despite the licensure of the pneumococcal conjugate vaccine (PCV) in the US and other Western countries for over 14 years, as of September 2014 only 4 South Asian countries were using PCV in their universal immunization program. To generate momentum toward addressing this issue a “South Asia symposium on pneumococcal disease and the promise of vaccines” was organized just prior to the 9th international symposium on pneumococci and pneumococcal diseases held in India recently. Leading scientists, program managers, and decision makers including ministry officials from the region participated in the meeting. The participants discussed available data on pneumococcal disease burden in South Asia, surveillance methods, efficacy and safety of pneumococcal conjugate vaccines (PCV), the status of PCV introduction, programmatic challenges in introducing PCV and available data on the impact of PCV in South Asia and globally. There was a strong consensus that available data on disease burden and the global experience with PCV justified the introduction PCV in all Asian countries in order to accelerate the gains in child survival in the region.

## 1 Introduction

The ninth international symposium on pneumococci and pneumococcal diseases (ISPPD-9) was held in Hyderabad, India from March 9 to 13, 2014. The ISPPD has been convening eminent scientists from around the world to discuss the latest scientific developments in the field of pneumococcal diseases and prevention strategies biennially since 1998. Previous ISPPD meetings paid special attention to the role of pneumococcal conjugates vaccines in disease prevention, emerging data on serotype replacement post-immunization, and the importance of continuous disease surveillance both before and after pneumococcal vaccine introduction [1], [2], [3]. ISPPD-9 reviewed advancements made in these areas, and also examined evidence that could inform the development of policies and programs relating to pneumococcal disease, and accelerate access to pneumococcal vaccines in the world's poorest countries.

ISPPD-9 also marked the first time, the conference was held in Asia. It provided an extraordinary opportunity to foster collaborations, promote knowledge sharing and examine topics ranging from the epidemiology of and diagnostics for pneumococcal disease to the development of disease control policies in low- and middle-income countries (LMICs). To this aim, a satellite meeting – the South Asia symposium on pneumococcal disease and the promise of vaccines – was held in conjunction with ISPPD on March 9, 2014. Leading scientists, program implementers, and decision-makers including officials of Ministries of Health from Asian countries convened to examine and review the available scientific data and share policy and programmatic considerations for pneumococcal conjugate vaccine (PCV) introduction. The purpose of the symposium was to accelerate the introduction and uptake of PCV in the region, specifically in countries like India facing the highest burden of pneumococcal disease.

The co-chairs of the South Asia symposium, Dr. Narendra Arora and Dr. Mathuram Santosham opened the meeting by remarking on the timeliness of the gathering, underscoring the need for tackling pneumonia as a top public health priority and highlighting the promising role of prevention through vaccination in reducing pneumonia-related childhood morbidity and mortality.

The key-note speaker, Dr. Rakesh Kumar, Joint Secretary (Reproductive & Child Health Programme) to the Government of India, Ministry of Health and Family Welfare shared India's progress toward improving child and maternal health, and reviewed the steps India had taken to reinvigorate its commitment toward achieving the millennium development goals (MDGs). Specifically, the national government had increased allocations from 1.36% to 1.87% of the GDP to the health sector and implemented policies to scale-up high-impact interventions for newborn and child survival, including key interventions to reduce pneumonia-related deaths in India [4], [5]. Specifically, Dr. Kumar acknowledged existing health system barriers – such as, the irregular supply of essential antibiotics, inadequate capacity of the public health workforce, and the failure to implement policies at the subnational level – that have stymied progress toward reducing pneumonia-related deaths [4]. In his concluding slide, Dr. Kumar reflected on the policy decision to introduce PCV currently facing the country [4].

This query set the stage for the symposium's agenda of examining available evidence to facilitate discussions and debate to identify barriers to PCV introduction and lessons learnt from other countries. This article presents a summary of these deliberations and the conclusions of the symposium according to the following thematic areas:

1. Epidemiology, disease surveillance methods, and estimates of pneumococcal disease burden globally and in South Asia.
2. Safety, efficacy, and the impact of PCV.
3. Policy and programmatic considerations to support country-level decision-making for PCV introduction.
4. A call to action to leverage the promising role of PCV in preventing pneumococcal disease burden in the region.

## 2 Burden of pneumococcal disease in South Asia and challenges of pneumococcal disease surveillance

The first panel reviewed the epidemiology of the pneumococcal disease globally and in South Asia and highlighted the successes and challenges of disease surveillance in Asia. Dr. Thomas Cherian presented World Health Organization's (WHO) unofficial estimates from 2008, which reported approximately 100,000 deaths from pneumococcal pneumonia and 13,000 deaths from pneumococcal meningitis in South Asia based on data from Bangladesh, Bhutan, India, Maldives, Nepal, Pakistan and Sri Lanka [6]. Dr. Cherian also described the challenges of establishing *Streptococcus pneumoniae* (*Spn* or pneumococcus) etiology and how it had limited the direct estimation of the pneumococcal disease burden. In particular, evidence supported that surveillance of laboratory confirmed culture positives of *Spn* disease represented only the “tip of the iceberg” because a vast majority of pneumonia cases are not easily diagnosed [6], [7]. Experts at the meeting generally agreed that the previously held belief of low pneumococcal disease burden in Asia was likely due to diagnostic factors like lowered sensitivity of culture techniques, antibiotic use before diagnostic work up, and delay in processing of specimens, which limited the availability of national estimates on disease burden. When newer diagnostic techniques were used, the rates of disease were similar to other developing regions of the world such as Africa [8]. For example, rates of invasive pneumococcal disease (IPD) detection were consistently higher in studies which used antigen detection techniques like latex agglutination and immunochromatography, or molecular assays like PCR in conjunction with culture especially for detection of pathogens in the cerebrospinal fluid [9], [10].

Furthermore, experts acknowledged the differences in the annual incidence of invasive pneumococcal disease (IPD) among five year olds living in countries sharing similar socio-economic and mortality profiles in the region. They concluded, the incidence of IPD among under five year olds – ranged from approximately 204 per 100,000 in Sri Lanka to 3058 per 100,000 in Bangladesh and that these differences were likely representative of differences in the diagnostic methodologies and surveillance methods that were used [11].

Based on the available epidemiological data reviewed at the symposium, experts at the meeting agreed there was sufficient evidence at the time to conclude the epidemiology of *S. pneumoniae* in Asian countries was comparable to that seen in other countries with similar socio-economic and mortality profiles prior to vaccine introduction. Therefore, in order to address the high burden of pneumococcal disease in region, particularly in India, Pakistan and Bangladesh, there was strong consensus to improve surveillance for invasive pneumococcal disease by providing adequate financial and technical support by global development partners. Although specifically not discussed, alternative and innovative financing mechanisms that have successfully expanding the fiscal space for health should be considered as potential tools for financing new vaccine introduction [12].

Dr. Pushpa Wijesinghe synthesized the successes and challenges of IPD surveillance from the experiences of the WHO-supported surveillance networks established in South and Southeast Asian countries. Establishing in-country sentinel surveillance networks had several advantages, including the generation of local and regional data on pneumococcal disease incidence and prevalence, as well as, bringing transparency to decision making processes at the National Immunization Technical Advisory Groups (NITAGs) in different countries [11]. The NITAGs in Bangladesh and Nepal selected PCV10 over PCV13 for national introduction in 2015. In making that decision, they took into account many factors including serotype coverage, presentation of the vaccine, cost of the vaccine, and cold chain issues etcetera. Similarly, regional data generated through these surveillance networks informed Myanmar's decision to introduce PCV10 despite lacking country-based surveillance mechanisms.

Evidence of IPD serotype distribution through surveillance networks also addressed the historic concerns about the serotype distribution in Asia, where questions about regional serotype coverage of the currently available pneumococcal vaccines have been raised. Available evidence on serotype prevalence for pneumococci in the region from India, Nepal, Bangladesh and Sri Lanka demonstrated that PCV10 covers approximately 70% and PCV13 covers approximately 74% of the serotypes which cause IPD [13]. These data are important for countries like India and Sri Lanka that are still determining whether to introduce PCV in their national immunization programs, as well as for countries conducting evaluations to assess the impact of PCV on both vaccine and non-vaccine type pneumococcal serotypes. It was also reported that IPD surveillance networks enhanced the capacity of country-led surveillance efforts and informed policy changes in certain countries. For example, India and Bangladesh integrated Acute Meningitis/Encephalitis Surveillance (AMES) with their existing polio and measles surveillance networks [11]. In addition, in Sri Lanka, data on antibiotic sensitivity gathered from pneumococcal surveillance informed changes in the antibiotic prescription policy [11].

Despite these successes, several concerns about the sustainability of these WHO-supported networks were raised, specifically, the dependence on external funding of ongoing surveillance activities, the lack of country leadership and the immediate need for establishing adequate surveillance systems in order to monitor serotype replacement in countries post-vaccine introduction [11]. Other challenges that were discussed related to diagnosis and data quality, for example, the lack of standardization of surveillance practices and data management, poor quality of existing culture techniques, limited access to newer higher sensitivity diagnostics, extensive use of antibiotics prior to obtaining diagnostic specimen, and difficulty in the timely transfer of specimens to reference laboratories [11].

Dr. Gungaa Surenkhand highlighted the challenges of surveillance for invasive bacterial diseases (IBD) caused by pneumococci in Mongolia. Results from six IBD surveillance sites in Mongolia showed low rates of etiologic diagnosis and illustrated the challenges experienced regionally in surveillance [14]. Unlike surveillance for measles and acute flaccid paralysis (AFP) for polio, conducting high quality facility-based surveillance of pneumococci required significant cooperation from clinicians, application of standardized case definition, as well as the implementation of specimen collection protocols and advanced laboratory techniques. To address some of the challenges, annual trainings on surveillance procedures were held for nurses and doctors, consultations were conducted to ensure compatibility between the surveillance case definition of pneumonia and the definition in Integrated Management of Childhood Illness (IMCI) guidelines, and the frequency of pneumococcal disease reporting was increased from the standard of twice per year to monthly. Despite some improvements, Dr. Surenkhand reported that the high rate of pre-hospital antibiotic use was a formidable barrier affecting laboratory testing and IBD surveillance in Mongolia. As of March 2015, Mongolia, a Gavi-graduating country was approved to receive support through advance market commitment for PCV introduction in 2016 [15].

With the introduction of PCV in countries in the region, panel participants concluded there was potential to leverage existing surveillance systems for important post-introduction impact evaluation studies and routine surveillance, however, in order to do so, countries would need to take ownership, and exert political pressure in order to enhance resources to support the establishment of additional surveillance sites as needed and strengthen existing systems.

## 3 Spotlight on pneumococcal conjugate vaccines

Dr. Rajesh Kumar provided an overview of the history, development, and efficacy of pneumococcal vaccines with a specific focus on PCVs, which is still currently under consideration for introduction in India. At the time, both PCV10 and PCV13 were licensed and available in the Indian private sector [16], but remained unaffordable to the masses and will continue to do so until the vaccine is introduced in the Indian National Immunization Schedule. Findings from a study reported that the high cost of PCV remained a major barrier for patients and limited access despite doctors’ recommendations to vaccinate [17]. To date, PCV has not been used in the public sector and also to our knowledge there is limited use of PCV in private sector with no reported coverage data in India. Because India has the highest number of pneumonia deaths in the world with pneumonia contributing to approximately 32% of the deaths in the 1–59 months age group in India^1^ [18], experts agreed that vaccines against pneumococci should be an integral part of a comprehensive approach to prevent pneumonia and reduce related child morbidity and mortality.

The available data estimates PCV efficacy at 80% (95%CI 58–90%) for vaccine serotypes, 58% for all serotypes, 27% for radiological pneumonia and 6% for clinical pneumonia [19]; Dr. Kumar suggested that India will need to leverage better diagnostic tools to monitor pneumococcal disease burden and conduct efficacy trials for PCV10 and PCV13 in order to mobilize a decision of vaccine type. Given available data indicating acceptable safety profiles of the vaccines and additional protective benefits of the vaccination (e.g., reduction in hospitalizations and potential to prevent deaths) [16], there was a strong consensus to consider the introduction of PCV in the National Immunization Schedule in India and enhance surveillance of the pneumococci serotypes pre- and post-vaccine introduction. One study also reported high coverage of PCV would result in significant cost savings to both the public and private sectors, especially in high mortality states such as Bihar, which face some of the highest burden of disease [20].

Dr. Cynthia Whitney presented evidence on the indirect effects of PCV. Indirect effects also known as herd immunity, refers to the reduction of pneumococcal disease in unvaccinated individuals who live in the same communities as vaccinated individuals. One study conducted in United States after the introduction of the heptavalent PCV (PCV7) in 2003 reported a higher (94%) reduction in vaccine type disease in children <5 years of age than expected (64%) based on the PCV coverage [21]. Indirect protective effects of PCV7 were also reported consistently across different populations in high income countries with vaccine coverage as low as 40% [22], [23]. Recently published data from studies in Kilifi, Kenya presented by Dr. Laura Hammitt at the symposium demonstrated two thirds decrease in nasopharyngeal carriage with vaccine type strains of *S. pneumoniae* in individuals of all age groups following programmatic use of PCV10 [24]. Other publications from Gambia and South Africa supported this finding [25], [26].

Though the magnitude and rate at which these indirect effects develop depend on vaccination coverage and proportion of disease caused by vaccine serotypes, evidence presented at the symposium indicated that indirect protective effects of vaccination resulted in disease reductions across unvaccinated age groups in both high- and low-income country settings. Therefore, experts agreed that indirect effects will likely be seen in Asian countries as the vaccine is introduced after 40–50% of the population is immunized. They also recommended for policymakers to factor in the benefits of indirect effects into cost effectiveness estimates and other analyses used in informing policy decisions concerning PCV introduction [22], [27]. Findings form one study in the United States, which incorporated the indirect effects of PCV7, demonstrated that the vaccine is not only cost effective but also leads to significant cost-savings [27].

Dr. Samir Saha also presented data on the potential role of PCVs in reducing the prevalence and spread of antibiotic resistant strains of pneumococci. The vaccines contain most of the serotypes associated with drug resistance, and studies reported that PCV use was associated with reduction in these strains in both the vaccinated and unvaccinated population [28]. There was also evidence that extensive use of PCV decreased the emergence of antibiotic resistant strains in the community by reducing antibiotic use [29]. While the possibility of serotype replacement with strains that could potentially become prone to drug resistance was raised, to date, no data has reported such a phenomenon [30]. Dr. Saha also highlighted the importance of post introduction surveillance, particularly in the context of monitoring serotype replacement and antibiotic sensitivity to guide the selection of future PCV vaccine candidates [30].

## 4 Regional experiences with the introduction of pneumococcal conjugate vaccines

In the final panel, experts shared their unique country-specific experiences relating to PCV introduction, presenting on various topics ranging from epidemiological data to policy-making and program implementation.

In October 2012, Pakistan became the first South Asian country to introduce PCV10 with Gavi support [31]. Dr. Asad Ali presented findings from the surveillance of pneumococcal meningitis among children in the Sindh province prior to PCV introduction, which was conducted with Gavi support. The study found a low rate of direct detection of *S. pneumoniae* but adjustments for case recruitment and diagnostic sensitivity showed Pakistan had rates of pneumococcal meningitis similar to those seen in higher income countries in the pre-vaccine era [32]. He also presented the objectives of the PCV10 evaluation study, which will assess the impact of PCV on IPD and nasopharyngeal carriage [33]. In addition, a Gavi-sponsored study of the impact of PCV 10 in Sindh (Pakistan) is ongoing with a focus on determining coverage rates and evaluating efforts to improve coverage in selected districts. Findings of the latter studies will be published in 2016 [33].

Dr. Eric Tayag presented the experiences of PCV10 introduction in 2013 in the Philippines [34], a non-Gavi country. Pneumonia was the leading cause of death among children under five years of age in 2010 and 2011. Professional organizations and advocacy groups, such as, the Philippine Foundation for Vaccination championed for PCV introduction particularly in the context of achieving MDGs and Universal Health Coverage. Due to the high vaccine price ($55 per child), the vaccine was introduced in a phased manner. Initially only 300,000 eligible infants in two regions with high burden of illness were targeted for vaccination with PCV 10 in 2013 and plans were made to vaccinate another cohort of 300,000 eligible children in four regions facing a high burden of disease in 2014. In 2014, PCV13 was selected based on a WHO Western Pacific Region study, which concluded that PCV 13 had higher cost–effectiveness when compared to PCV10.

The Philippines addressed several barriers and created an enabling environment for PCV introduction by strengthening IPD surveillance, designing impact studies at low cost, and demonstrating the benefits of vaccines [34]. They also accessed vaccines from cheaper markets and engaged development and advocacy partners in order to continue efforts to improve child health and survival [34].

Dr. Anonh Xeuatvongsa reported on the phased introduction of PCV13 in Lao PDR in 2013 [35], [36]. The national government recognized that respiratory infections were the leading cause of morbidity and mortality among children under five years of age in Lao PDR. Rural communities in particular had poor access to antibiotics and demand for an effective vaccine was high, especially among parents who had seen their children suffer from the symptoms. Though the only data available from a major urban hospital showed low IPD rates as diagnosed by culture, evidence from studies that incorporated more advanced diagnostic techniques demonstrated higher burdens of pneumococcal disease in Asia similar to pre-vaccine global rates. There was strong consensus among decision-makers that pneumococci contributed to a large portion of severe pneumonia and was a major cause of child illness and death. Furthermore, efficacy and the benefits of PCV impact, which had been sufficiently demonstrated globally, solidified Lao PDR's decision to introduce the PCV into the national immunization program. The vaccine was introduced in the capital Vientiane and Vientiane province with Gavi support in 2013 and at the time, a tentative nationwide roll-out was planned for 2014. Now, Lao PDR faces the challenge of measuring PCV impact since accurate baseline data are not available [36]. The country is collaborating with international academic centers and development partners to support such evaluation studies.

## 5 Call to action for the introduction of PCV and concluding remarks

Based on the wealth of data presented demonstrating the evidence of the burden of disease and the positive impact of PCV, the symposium concluded that countries should prioritize introducing PCV through national immunization programs as an integral part of a comprehensive strategy to prevent pneumonia and improve child health. Emphasis was placed on India where PCV introduction is expected to not only lower the incidence of pneumonia but also address the substantial burden of invasive pneumococcal disease. Data on PCV effectiveness in LMICs in Asia and Africa also demonstrated the potential for PCV to have a large impact in reducing deaths and hospitalization among the poorest children who face the highest risk of disease and death. At the same, the symposium delegates acknowledged that critical gaps exist for implementing an effective PCV introduction and evaluation program. These limitations range from diagnostic challenges of estimating disease burden, availability of well performing surveillance sites and health system barriers which can severely affect vaccine delivery and the ability to conduct impact evaluation. With country-level leadership, adoption of new diagnostic techniques, the expansion of laboratory surveillance and strengthening of health systems capacity, the introduction of PCV will provide an essential tool to reduce child mortality accelerate the gains in child survival for the region. The symposium participants made a strong plea to all countries in Asia to make the introduction of PCV a top priority in their respective countries in order to save countless lives in the next decade.

## Funding

The symposium was sponsored by GlaxoSmithKline, Pfizer and the Bill & Melinda Gates Foundation.

## Footnotes

1 Calculated using data from Liu et al. (2012).
